# Impact of different omega-3 fatty acid sources on lipid, hormonal, blood glucose, weight gain and histopathological damages profile in PCOS rat model

**DOI:** 10.1186/s12967-020-02519-1

**Published:** 2020-09-14

**Authors:** Fiza Komal, Muhammad Kamran Khan, Muhammad Imran, Muhammad Haseeb Ahmad, Haseeb Anwar, Usman Ali Ashfaq, Nazir Ahmad, Amna Masroor, Rabia Shabir Ahmad, Muhammad Nadeem, Mahr Un Nisa

**Affiliations:** 1 Institute of Home and Food Sciences, Faculty of Life Sciences, Government College University, Faisalabad, Punjab Pakistan; 2 Department of Physiology, Faculty of Life Sciences, Government College University, Faisalabad, Punjab Pakistan; 3 Department of Bioinformatics and Biotechnology, Faculty of Life Sciences, Government College University, Faisalabad, Punjab Pakistan; 4 Department of Dairy Technology, University of Veterinary and Animal Sciences, Lahore, Punjab Pakistan

**Keywords:** PCOS, Infertility, Ω-3 fatty acids, Flaxseed oil, Fish oil

## Abstract

**Background:**

Omega-3 fatty acids (Ω-3 PUFAs) may help to improve health status in polycystic ovarian syndrome (PCOS) by reducing numerous metabolic disorders (insulin sensitivity, hyperinsulinemia, lipid profile, obesity and inflammation). To evaluate the current objective, 16 weeks (6 weeks of adjustment period followed by 10 weeks of collection period) research trial was planned to check the impact of different sources of Ω-3 PUFAs (synthetic Ω-3, flaxseed and fish oil) on nutrient digestibility, weight gain, productive (lipid profile, glucose and insulin), reproductive profile (progesterone, follicle stimulating hormone (FSH), estrogen, luteinizing hormone (LH) and prolactin) and histological study of ovarian tissues in Wistar female rats.

**Methods:**

Forty-five rats of 130 ± 10 g weight were divided into 5 groups, each having 9 rats: NC (negative control without PCOS), PC (positive control with PCOS), SO (synthetic omega-3 containing ALA, EPA and DHA), FO (flaxseed oil) and F (fish oil) fed at 300 mg/kg/orally/daily of these sources were added in the basal diets while PC and NC received only the basal diet. Food and water were offered ad libitum. PCOS was induced in the rats fed of PC, SO, FO and F diets group by single intramuscular injection of estradiol-valerate (4 mg/rat/IM). Body weight and blood glucose was recorded weekly. At 16^th^ week of trial, blood samples were collected for lipid and hormonal analysis. Ovarian tissues were removed for pathological evaluation. Digestibility was measured by total collection method.

**Results:**

Cholesterol, triglycerides and low-density lipoproteins were reduced in SO, FO and F groups when compared with rats of PC group. However, increasing trend of high-density lipoprotein (HDL) was found in same groups. The highest HDL (36.83 ± 0.72 mg/dL) was observed in rats fed F diet. In case of a hormonal profile, testosterone, LH and insulin levels showed a significant reduction after treatments. Blood glucose results showed significantly reducing trend in all the rats fed with Ω-3 PUFAs sources than PC from 5 to 10th week of trial. However, similar trend was noticed in rat’s body weight at the end of 6th week. In ovarian morphology, different stages of follicles were observed in groups fed SO, FO and F diets. Nutrient digestibility in PCOS induced rats was remained non-significant.

**Conclusions:**

The three sources of Ω-3 PUFAs had effective role in improving lipid and hormonal profile, reducing blood glucose, weight gain and histopathological damages in PCOS rats. However, fish oil source might be an innovative approach to cure PCOS via reducing the weight and metabolic anomalies due to EPA and DHA.

## Background

Polycystic ovarian syndrome (PCOS) is the endocrine disorder mainly occurs in 4–10% at reproductive age due to nutritional imbalance [1]. Imbalance in nutrient intake induces several metabolic diseases (dyslipidemia, insulin resistance and obesity) that will also lead to imbalance reproductive hormones (androgen, progesterone, estrogen, prolactin, FSH and LH) and ultimately causes the PCOS [2]. In obese females, inflammation [3] and insulin resistance encourage the ovarian dysfunction for the androgen production [4]. In PCOS therapeutic management, medicine like metformin has been used conventionally. Dietary intervention for the reduction of obesity, dyslipidemia and hyperinsulinemia in PCOS patients is limited. That’s why omega-3 fatty acid (Ω-3 PUFAs) sources were selected as dietary intervention to synchronize the irregular metabolite in PCOS rats.

Several clinical and experimental researches showed that Ω-3 PUFAs have been used for improving the lipid and insulin profile in obesity and inflammation [5–7]. However, responses of various food sources of Ω-3 PUFAs are different due to variation in its chemistry configuration. Two food sources, flaxseed {α-linolenic acid (ALA)} and fish oil {(eicosapentaenois acid (EPA), docosahexaenoic acid (DHA)} contain chemically different Ω-3 PUFAs. Very few researchers have observed that which important food sources with their Ω-3 PUFAs profile had impact to reduce the metabolic disorder in induced PCOS rats. The goal of this study was to confirm the impact of dietary and synthetic sources of Ω-3 PUFAs by which they can modify lipid and hormonal profiles, glucose level and body weight in PCOS induced rat model.

## Methods

### Procurement of rats and their administration

Forty-five days old, weighing 130 ± 10 g Wistar Albino female rats, which have 2 repeated estrus cycles had been bought from National Institute of Health (NIH), Islamabad, Pakistan. They were kept in a room under 12 h light/dark cycle, at 25 ± 1 °C temperature and relative humidity of 45 to 55%. All experimental protocols for rats were carried out by adopting the procedures for the precaution and usage of laboratory animals accepted by the National Institutes of Health guide (NIH Publications No. 8023, reviewed 1978). Animal Ethical Committee of Government College University, Faisalabad, Pakistan had proved this research by adopting Principles of Laboratory Animal Care. Completely Randomized Design was used by dividing rats into five groups each having 9 rats: NC (Negative control without PCOS), PC (Positive control with PCOS), SO (synthetic omega-3 containing ALA, EPA and DHA), FO (flaxseed oil) and F (fish oil) fed at 300 mg/kg/orally/daily of these sources were added in treatment groups by replacing that amount of fat content in basal diet, while PC and NC received only the basal diet (Table 1) [8]. Treatment groups were established by a balanced random allocation scheme. *Isocaloric* and *isonitrogenous* diet was prepared for all groups according to National Research Council (NRC) [9]. Animal feed and water was given ad libitum*.* The duration of in vivo trial was 16 weeks from which 6 weeks were adjustment period and 10 weeks were collection period. Food intake by the experimental rats was calculated weekly as the difference noted in the amount of food given, amount consumed and the amount remaining in each cage after 1 week of experimental period. PCOS were induced in all groups except NC by single intramuscular injection of estradiol-valerate (4 mg/rat/IM) dissolved in 0.2 mL oil during acclimatization period [10]. Subsequently, the PCOS induction was verified by visual method [11] and regular analysis of vaginal smear during anovulatory estrous cycle was performed during the examination of the study period [12]. Blood glucose and weight gain were recorded weekly.

**Table 1:** Composition of experimental diet

Ingredients (g/1000 g diet)	NC	PC	SO	FO	F
Cornstarch	347.49	347.49	347.49	347.49	347.49
Maltodextrin	132	132	132	132	132
Sucrose	100	100	100	100	100
Casein	200	200	200	200	200
L-Cysteine	3	3	3	3	3
Soyabean oil	70	70	50.01	32.6	16
Cellulose	100	100	100	100	100
AIN-93-VX vitamin mix	10	10	10	10	10
AIN-93G-MX mineral mix	35	35	35	35	35
TBHQ	0.014	0.014	0.014	0.014	0.014
Choline bitartrate	2.5	2.5	2.5	2.5	2.5
SO	0	0	19.99	0	0
FO	0	0	0	37.40	0
F	0	0	0	0	54
Total energy (kcal)^a^	3660	3660	3660	3660	3660

NC, Negative control; PC, Positive control; SO, (synthetic omega-3 containing ALA, EPA and DHA); FO, (flaxseed oil); F, (fish oil) and TBHQ, tertiary-butylhydroquinone

^a^The estimate of caloric content was based on the standard physiological fuel values for CHO, fat and

protein of 4, 9 and 4, respectively

### Fatty acids composition of oils by gas chromatography (GC)

Preparation of reagents and methyl esters of fatty acids in the experimental oil samples under study were performed according to the method described by Asghar and Majeed [13] with some modifications. 2 mL of methanolic H_2_SO_4_ was mixed with 0.2 mL oil in a 50 mL screw capped Pyrex glass tubes. These glass tubes were put in a pre-heated oven at 80 °C. The time was fixed for 1 h and frequent shaking of the tubes was carried out after 15 min. Then, these glass tubes were taken out, cooled to room temperature and 2 mL of distilled water were added in each tube to stop the esterification reaction. After the completion of esterification process, the fatty acids esters were extracted using the petroleum ether (1 mL). This process was repeated thrice. After that, the ether content was evaporated under inert gas in heating block and remaining darkish oily upper surface (1 μL) was injected into gas chromatograph (Agilent Technologies Inc. USA) equipped with flame ionization detector (FID) and column to obtain fatty acid methyl ester peaks. The column temperature was set at 150 °C and detector temperature was standardized at 250 °C. The temperature was increased at the rate of 10 °C/minute to 250 °C and held there exact for 5 min. The total experimental run time was noted 45–50 min for each sample. The individual peaks for all fatty acid methyl esters were identified. The obtained retention times of experimental peaks were compared with standard peaks. The fatty acid composition against each peak was calculated using the peak areas of the fatty acid species that appear in the chromatogram.

### Lipid profile analysis

Lipid profile was checked by micro plate reader URIT 660 (URIT Medical Electronic Co., Ltd, Guangxi, China). Cholesterol was determined with kit method using Biosystems cholesterol kit REF. 11,505 (Barcelona, Spain) having limit of detection 4.2 mg/dL (0.10 mmol/L) and linearity limit (1000 mg/dL; 26 mmol) with inter- and intra-assay CV 1.1%. Triglyceride was estimated by Triglycerides kit (BioSystems S.A., Barcelona, Spain). The detection limit was 4.4 mg/dL (0.05 mmol/L) and linearity limit (600 mg/dL; 6.78 mmol) while inter- and intra-assay CV was 2.8%. LDL was determined through Wiener kit REF. 1,220,229 (Rosario, Argentina) having CV in the range of 2.6%. The detection limit was 0.4 mg/dL (0.01 mmol/L) while linearity limit was detected 1000 mg/dL (26 mmol). HDL was determined through Wiener kit REF 1,220,114 (Rosario, Argentina) with CV in the range of 3.8%. The detection limit was 0.5 mg/dL (0.01 mmol/L) while linearity limit was noted 200 mg/dL (5.18 mmol) [14].

### Hormonal profile analysis

Testosterone, progesterone, estrogen, follicle stimulating hormone (FSH), prolactin and luteinizing hormone (LH) were estimated through Enzyme Linked Immunosorbent Assay (ELISA) kits method (Biocheck, Inc. Foster City, CA 94,404, USA). Insulin was analyzed via ELISA kit method (Monobind Inc. Lake Forest CA 92630 USA).

### Blood glucose

Weekly Blood glucose was checked by using the Accu-Chek® Active glucometer (Roche Diagnostics GmbH, Germany).

### Body weight

Body weight of all rats were measured weekly by weighing balance.

### Histological study of ovarian tissues

The ovarian tissues were put in buffer formalin (10% solution) and fixed firmly in paraffin wax. 5-micron thick part (slice) was taken and staining was performed using eosin and haematoxylins solution. The cross sections (total 100 in number) per specimen were checked by using light microscope (Olympus, 3H-Z-Japan) to diagnose hyperaemia [10].

### Blood sampling

At the last day of the study, 5 mL blood samples of rats were obtained for analyzing the serum lipid, insulin and reproductive hormone profile. Ovarian tissues were removed for pathological evaluation [10].

### Nutrient digestibility

The nutrient digestibility assay was determined using quantities of nutrient intake and total produced excreta. Nutrients intake was recorded weekly until the end of trial while last ten days were kept for feces collection. The collected excreta (500 g) were homogenized for posterior removal and were placed in a forced ventilation oven at calibrated temperature (60 °C) for calculated time (72 h) to get air-dried samples. Then, air-dried samples were grinded and weighed. Subsequently, the nutrient composition, dry matter, crude protein and gross energy profile of air-dried and experimental diets samples was determined to calculate the nutrient digestibility [15]. Based on the results of these parameters, the nutrient digestibility of the diets was calculated using the following expression.

$${\text{Nutrient Digestibility }}\left( \% \right) \, = \frac{{{\text{Nutrient intake }} - {\text{ Nutrient in feces }} \times {1}00 }}{{\text{Nutrient intake}}}$$

### Statistical analysis

Completely Randomized Design was used by making 5 experimental units, each treatment comprising of 9 rats. All the data collected were statistically measured for mean and standard error. Statistical analysis was carried out by one-way ANOVA using SPSS Statistics for Windows, version 17 (SPSS Inc., Chicago, Illinois., USA). Comparison of means for omega fatty acids doses on weekly blood glucose and body weight change was observed via Duncan Multiple Range Test [16]. The difference in the mean values of the two groups was considered statically significant if the “P” value was equal to or less than 0.05 and non-significant (N.S.) if the “P” value was greater than 0.05.

## Results

### Lipid profile

**Fig. 1:**
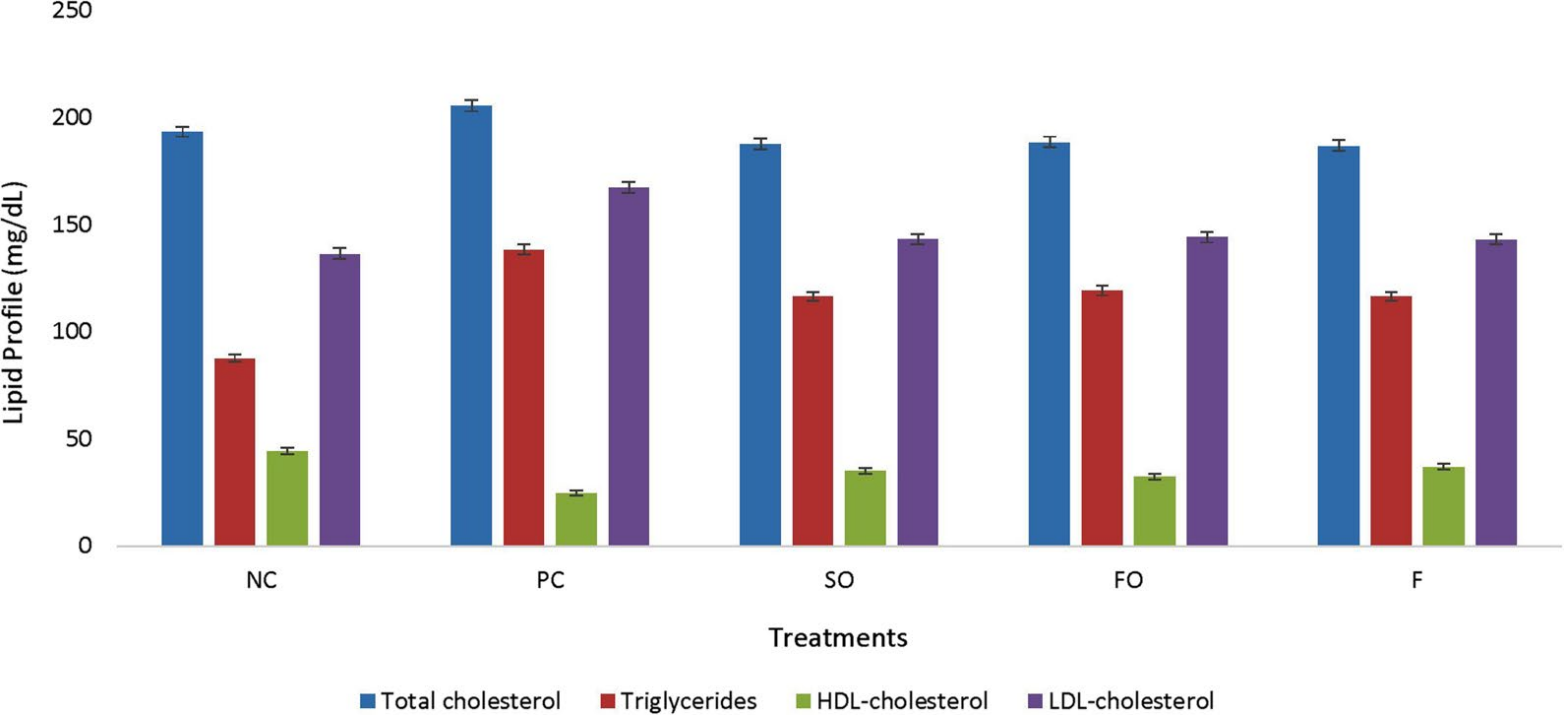
Effect of different sources of Ω-3 fatty acids on lipid profile in PCOS induced rats (negative control, NC; positive control, PC; synthetic Ω-3, SO 300 mg/kg/orally/daily; flaxseed oil, FO 300 mg/kg/orally/daily; fish oil, F 300 mg/kg/orally/daily; HDL, high density lipoprotein and LDL, low density lipoprotein)

The fatty acids composition of oils present in different experimental diets has been described comprehensively in Table 2. There was significant difference in lipid profile of rats in different groups, data is shown in Fig. 1. HDL in rats was increased 33.02, 29.37 and 23.81%, respectively in the rats of F, SO and FO groups when compared it with rats fed PC diet. However, cholesterol, triglycerides and LDL were significantly reduced in rats fed F, SO and FO diet as compared to rats fed PC diet. Highest reduction in serum cholesterol, triglycerides and LDL were observed in rats fed F diet that is about 16.28, 15.80 and 14%, respectively, when compared it with rats fed PC diet. However, overall trend in serum lipid profile was remained non-significant among rats fed SO, FO and F diets and become in normal range as shown in rats fed NC diet.

**Table 2:** Fatty acids composition of oils present in different experimental diets

Fatty acids g/100 g	Soybean oil	Flaxseed oil (ALA)	Fish oil (EPA + DHA)	Synthetic n-3 PUFAs (ALA + EPA + DHA)
Polyunsaturated fatty acids	57.9	70.35 g	22.5 g	100 g
Linoleic acid (18,2, n-6)	54.2	17.04	0.935	0
Linolenic acid (18:3, n-3)	7.7	53.31	0.935	33.33
EPA (20:5, n-3)	0.1	0	6.9	33.33
DHA (22:6, n-3)	0.1	0	11.97	33.33
Others	-	0	1.76	-
Monounsaturated fatty acids	22.78	20.19 g	46.7 g	-
Oleic acid (18:1)	20.4	20.19	2.81	0
Others	2.38	0	43.89	0
Saturated fatty acids	15.65	9.46 g	30.8 g	-
Myristic acid (14:0)	0.1	0.06	5.07	0
Palmitic acid (16:0)	9.7	5.30	18.6	0
Stearic acid (18:0)	3.6	4.10	2.7	0
Others	-	0	4.43	0

ALA, α-Linolenic acid; EPA, Eicosapentaenoic acid; DHA, Docosahexaenoic acid

### Hormonal profile

**Fig. 2:**
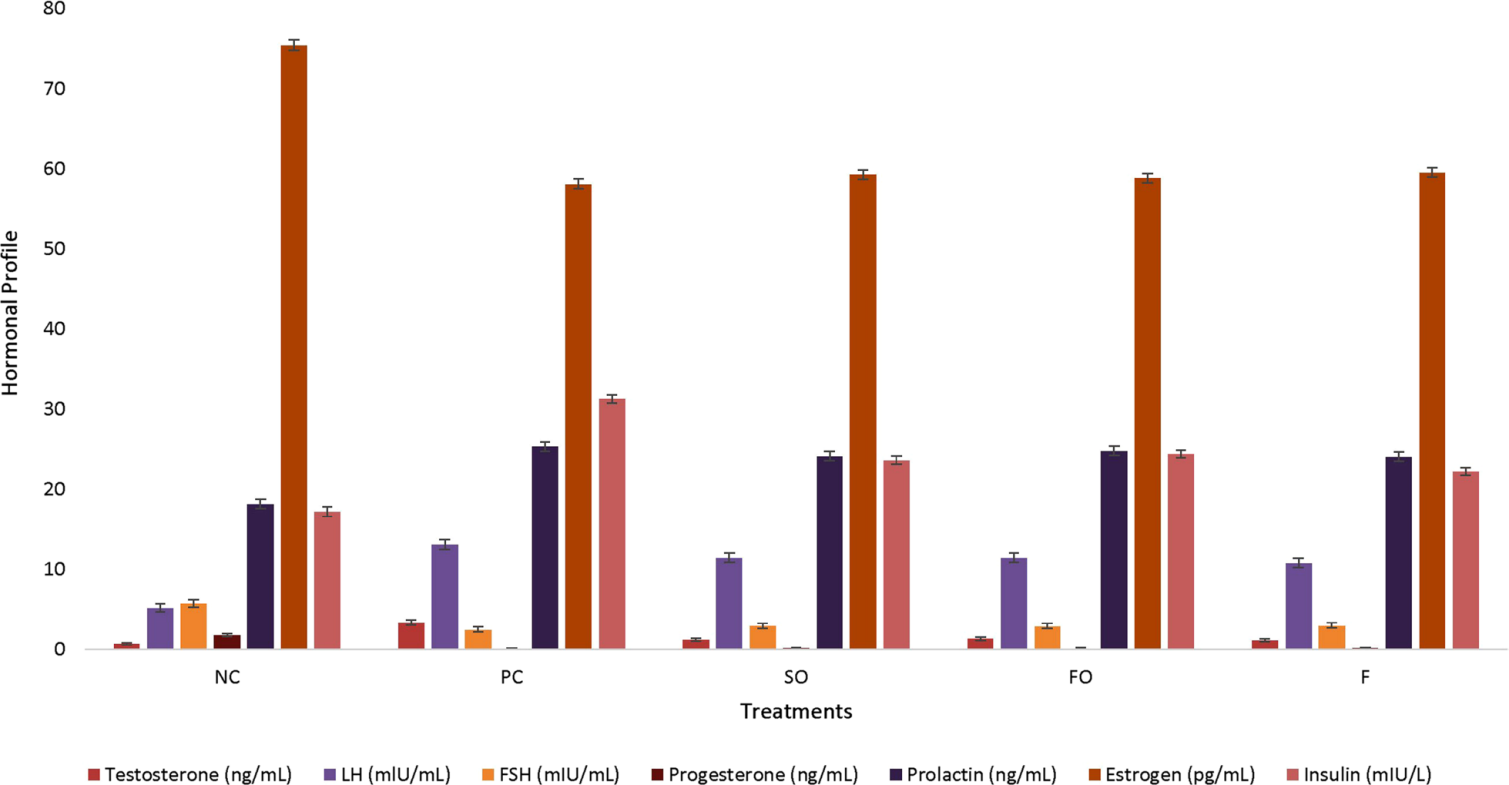
Effect of different sources of Ω-3 fatty acids on hormonal profile in PCOS induced rats (NC, negative control; PC, positive control; SO, synthetic Ω-3 300 mg/kg/orally/daily; FO, flaxseed oil 300 mg/kg/orally/daily; F, fish oil 300 mg/kg/orally/daily; FSH, follicle stimulating hormone and LH, luteinizing hormone)

The results regarding hormonal profile among rats fed NC, PC, SO, FO and F diets have been shown in Fig. 2. Results showed that after the supplementation of different sources of Ω-3 fatty acids, the levels of testosterone, LH and insulin were significantly reduced in rats fed SO, FS and F diets. However, all treatments were non-significant to each other and were significant from the PC. Serum progesterone, LH, prolactin and estrogen level in rats fed SO, FO, F and PC diets showed non-significant results.

### Blood glucose

At the end of 4^th^ week, SO, FO and F diets did not show any significant effect in rats blood glucose (Table 3). From 5 to 10th weeks SO, FO and F diet had positive effect on decreasing rat’s blood glucose that is about 17.03% reduction in last week when compared it with rats fed PC diet. However, this decreasing trend in rat’s blood glucose in SO, FO and F group was remained non-significant to each other.

**Table 3:** Effect of different sources of Ω-3 fatty acids on weekly blood glucose (mg/dL) in PCOS induced rats

Weeks	Treatments				
NC	PC	SO	FO	F	
1st	97.03 ± 0.73a	164.27 ± 0.64b	162.70 ± 0.90b	163.10 ± 0.81b	162.27 ± 0.92b
2nd	99.17 ± 0.60a	163.94 ± 0.91b	162.50 ± 0.98b	162.82 ± 0.98b	162.36 ± 0.95b
3rd	95.03 ± 0.88a	157.00 ± 0.57b	155.80 ± 0.64b	156.00 ± 0.66b	155.13 ± 0.62b
4th	91.82 ± 0.99a	160.73 ± 0.94b	158.23 ± 0.95b	158.70 ± 0.96b	157.73 ± 0.84b
5th	93.88 ± 0.92a	157.85 ± 0.64c	150.36 ± 0.73b	150.63 ± 0.79b	149.40 ± 0.97b
6th	85.06 ± 1.05a	159.32 ± 1.27c	145.46 ± 0.78b	146.00 ± 0.58b	145.77 ± 0.98b
7th	96.79 ± 0.67a	162.39 ± 0.39c	149.10 ± 0.81b	149.77 ± 0.27b	148.80 ± 1.11b
8th	92.83 ± 0.85a	163.47 ± 0.48c	140.87 ± 0.61b	141.10 ± 1.07b	140.47 ± 0.79b
9th	98.53 ± 0.74a	167.03 ± 0.73c	136.80 ± 0.36b	137.20 ± 0.89b	136.63 ± 1.22b
10th	99.26 ± 0.77a	165.84 ± 0.74c	137.50 ± 0.78b	139.77 ± 0.12b	137.60 ± 0.30b

PCOS, Polycystic Ovarian Syndrome

Negative control (NC); Positive control (PC); Synthetic Ω-3 (SO 300 mg/kg); Flaxseed oil (FO

300 mg/kg); Fish oil (F 300 mg/kg)

Within a row means denoted by a different letter are statistically significant (p < 0.05)

### Body weight

**Fig. 3:**
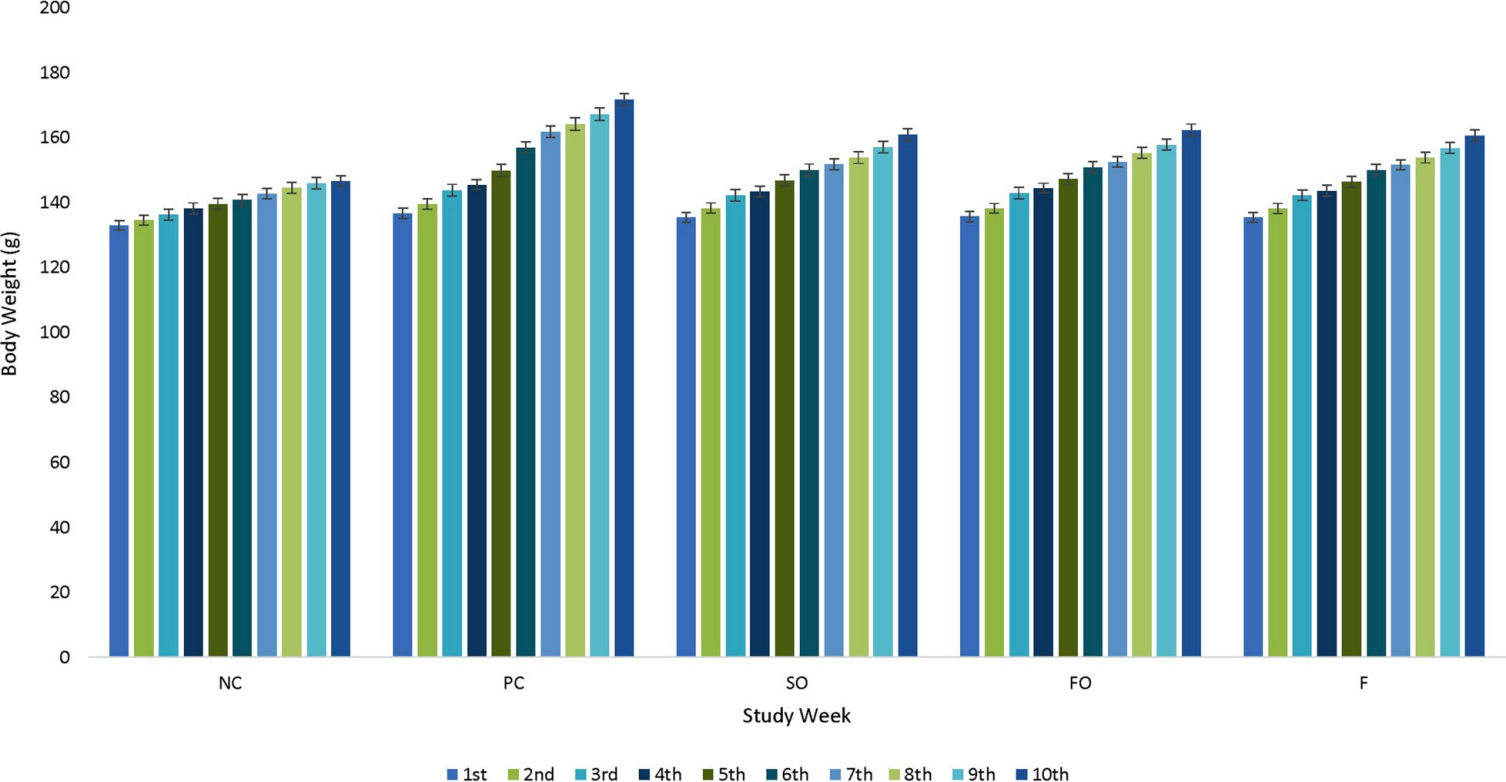
Effect of different sources of Ω-3 fatty acids on weekly body weight (g) in PCOS induced rats (negative control, NC; positive control, PC; synthetic Ω-3, SO 300 mg/kg/orally/daily; flaxseed oil, FO 300 mg/kg/orally/daily and fish oil, F 300 mg/kg/orally/daily)

At the end of 6th week, there was significant reduction in body weight of rats fed SO, FO and F diet as compare to rat’s body weight fed PC diet (Fig. 3). However, in the initial five weeks, all diets showed same trend in body weight of rats fed SO, FO, F and PC diets. At the end of trail, there was 11.07 g reduction of body weight observed in rats fed F diet than rats fed PC diet that is about 6%. Figure 3 shows that body weight of rats was controlled in group SO, FO and F groups as compared to PC that was increasing with age.

### Pathological evaluation of ovaries

**Fig. 4:**
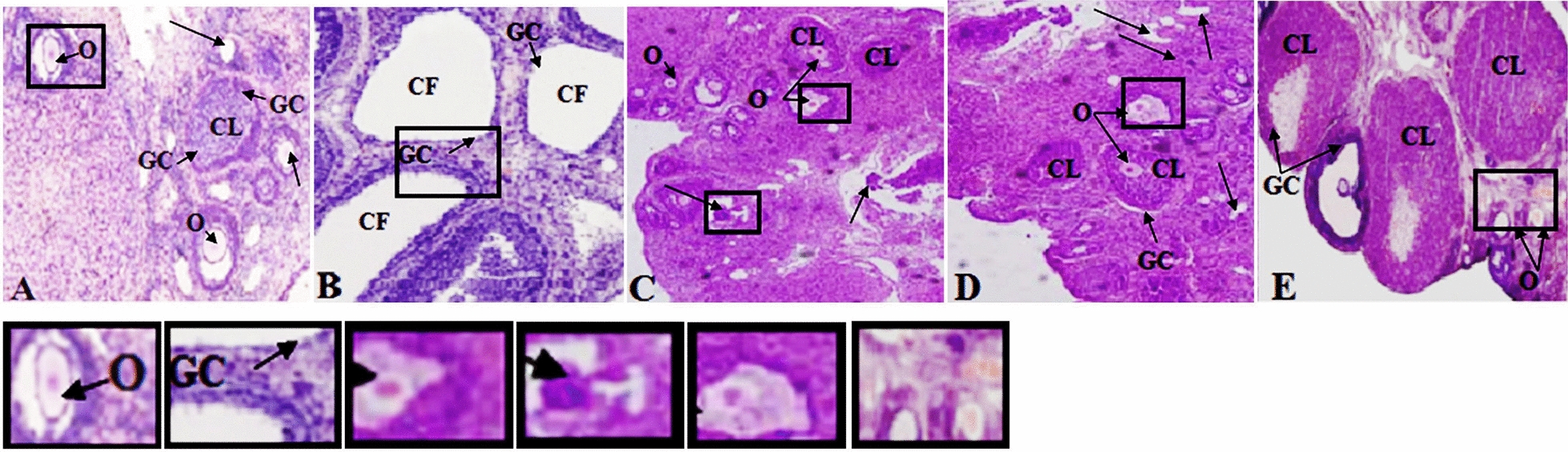
Ovarian histopathological examination of PCOS induced rats. Histological sections were stained with hematoxylin and eosin. In negative control group (**a** H&E × 100), various stages of follicles were developed normally with aggregation of granulosa cells. In positive control group (**b** H&E × 100), many follicular cysts were present in PCOS rats ovaries with degrading and thin layer of granulosa cells. After treatment of different sources of omega-3 fatty acids, few follicles with different developmental stages were observed in groups treated with SO, FO and F diets (**c**, **d**, **e**: H&E × 100). CF: (Cystic follicles), CL: (Corpus luteum), GC: (Granulosa cells), O: (Corpus Luteum).

Sections of ovaries from control group animals showed healthy structures of follicles in different development stages with aggregation of granulose cell (GC) and the corpus luteum (CL) (which is a definite sign of ovulation) were observed (Fig. 4a). A large number of cystic follicles (CF) with thin layer of granulose cells (GC) were observed in PCOS induced rats. Corpora lutea were completely absent indicating anovulation (Fig. 4b). After supplementation of different sources of Ω-3 fatty acids, no ovarian cysts were seen and normal sized healthy follicles at different developmental stages with oocytes, corpus luteum (CL) and clear, visible granulosa cell (GC) layer were observed in rats fed SO, FO and F diets (Fig. 4c–e).

### Nutrient digestibility

The results showed that the effect of different sources of Ω-3 fatty acids on nutrient digestibility in PCOS induced rats was remained non-significant. The digestibility of dry matter (DM), crude protein (CP), ether extract (EE) and crude fiber (CF) was not change statistically in all rats fed diets containing Ω-3 fatty acids sources compared with NC and PC. The results of nutrient digestibility of rats fed NC, PC, SO, FO and F diets are given in Table 4.

**Table 4:** Effect of different sources of Ω-3 fatty acids on nutrient digestibility in PCOS induced rats

Parameters %	Treatments				
	NC	PC	SO	FO	F
DM	75.40 ± 1.16	74.46 ± 0.81	75.27 ± 1.05	74.23 ± 1.31	74.98 ± 1.45
CP	74.26 ± 1.08	74.06 ± 1.00	73.56 ± 1.25	73.94 ± 1.39	74.34 ± 1.09
EE	86.54 ± 1.47	85.68 ± 1.25	85.13 ± 1.17	84.34 ± 1.16	84.23 ± 1.17
CF	36.42 ± 1.23	37.63 ± 1.16	38.12 ± 1.18	38.78 ± 1.32	38.12 ± 1.11

Negative control (NC); Positive control (PC); Synthetic Ω-3 (SO 300 mg/kg); Flaxseed oil (FO

300 mg/kg); Fish oil (F 300 mg/kg); Dry matter (DM); Crude protein (CP); Ether extract (EE) and Crude

fiber (CF)

DM, Dry matter; CP, Crude protein; EE, Ether extract and CF, Crude fiber

## Discussion

The current study confirmed that supplementation of Ω-3 PUFAs sources had healthy impacts on lipid profile, blood glucose and body weight of PCOS rats when compared it with rats fed PC diet. Numerous studies have found that feeding fish oil (FO) and flaxseed oil (FSO) helps to decrease cholesterol, triglycerides (TG) and LDL while increases the level of HDL in animal/human [17–20]. These food sources having Ω-3 PUFAs with different configuration named EPA, DHA and ALA play a key role for reducing the plasma TG, LDL and cholesterol by activating the transcription factors which control and regulate the nutrient traffic of lipid metabolism pathways in a tissue by enhancement of 5′ AMP-activated protein kinase (AMPK). The AMPK have been characterized as a major sensor of cellular energy status which furthermore regulates the partitioning process between lipid oxidation and lipogenesis [21, 22]. Various mechanisms have been prescribed in the literature to explain the effects of Ω-3 PUFAs on serum lipids. Ω-3 PUFAs slow down hepatic production of fatty acids in the biological system. The process mechanism may involve suppressing of gene expression of sterol regulatory element-binding proteins. As a result, the gene expression of enzymes is depressed which are involved in synthesis of fatty acids (fatty acid synthetase complex and acetyl-coenzyme A carboxylase) [20]. In the modulation of levels of triglycerides, Ω-3 PUFAs also influence additional nuclear receptors that are concerned with these e.g., farnesol X receptor, liver X receptor and hepatocyte atomic factor 4α. Furthermore, lipoprotein lipase is helpful for disposal of triglycerides from circling chylomicron particles [23]. However, some studies showed that EPA and DHA perform better as compare to ALA for altering the lipid profile because some people could not convert ALA in to active form for their proper functioning and utilization in the body [24].

The present research work has demonstrated that SO, FO and F had significant effect in reducing testosterone, LH and insulin while FSH, progesterone, prolactin and estrogen remained non-significant. The outcomes of recent research work are in line with the results of Azadeh et al. [25] who showed that after 8 weeks of the trial, the supplementation of Ω-3 PUFAs decrease the testosterone concentration. This decrease might be due to the effect of Ω-3 on LH. Hyperandrogenism has been observed as first clinical symptom in the patients with irregularity in steroid production. Generally, the increase in estrogen secretion leads to production of this condition. Furthermore, the LH hypersecretion is the major reason for increase in serum levels 17 hydroxyprogesterone and testosterone [26]. According to Phelan et al. [27], PUFAs supplementation leads to significant reduction in testosterone production level. It is also evident from the results that the concentrations of reproductive hormones like estrogen, LH, or FSH were not changed significantly. The previous studies reported that flaxseed consumption and/or supplementation in the experimental diets is effective in reducing the LH levels [28, 29]. LH has key role in androgen production and theca cells are responsible to produce the androgens in abundant concentration. Moreover, LH increases the production level of androgen hormones such as testosterone from the adrenal gland and ovary in PCOS which causes the infertility in the subject. The aromatization of these androgens to oestradiol is done by the FSH in granulosa cells and these conditions produce high number of immature follicles which is further enhanced by irregular production of androgen in PCOS. In this context, the omega-3 fatty acids help to decrease serum androgen levels [30].

Azadeh et al. [25] confirmed that after the addition of Ω-3, prolactin, progesterone and estrogen concentrations did not significantly change. In this case, no similar study of changes in prolactin has been found. The mean change in prolactin was non-significant after eight weeks of research. Women with PCOS are at risk of metabolic disorders including inflammation, hyperinsulinemia and oxidative stress [31]. There were only a few researches to estimate the effects of Ω-3 PUFAs in PCOS women on insulin. Mohammadi et al. [32] which confirmed that taking Ω-3 PUFAs from fish oil for eight weeks at a dose of 4 g/day had a positive effect on the level of serum insulin and glucose uptake in PCOS patients. EPA and DHA containing Ω-3 PUFAs have anti-obesity, anti-insulin resistance and anti-inflammatory functions [33] by enhance insulin sensitivity due to declining inflammatory cytokines including tumor necrosis factor, interlukin-6 and enhancing emission of anti-inflammatory adiponectin [34]. However, studies with comprehensive experimental time duration are suggested in order to get more effective results.

Blood glucose level was reduced (17–17.03%) in PCOS rats fed different sources of Ω-3 PUFAs at the end of 10^th^ week of trial when compared it with rats fed PC diet. Sahar [35] had also found similar result that there was 49.09%, 44.0% and 44.9% reduction in blood glucose level after offering supplementation of fish oil, flaxseed oil and fish oil + flaxseed oil, respectively at 8^th^ week of trial. The findings of different scientists who studied that supplementation of different sources of Ω-3 PUFAs have beneficial effects on glucose [36–39]. The Ω-3 PUFAs might be the important source for the reduction of blood glucose level by raising the sensitivity of insulin signal that attributed by receptor of G-protein for the production of glucagon-like peptide 1 (GLP-1) from enteroendocrine cells, up-regulation of the apelin pathway, and down-regulation of other control pathways for insulin production [40, 41]. In current study, the reduction in blood glucose noticed in PCOS rats fed different sources of Ω-3 PUFAs might be due to presence of EPA form that is important substrate for the formation of G-protein-coupled receptors [42]. It has also been observed that omega-3 fatty acids also enhance the function of adiponectin hormone which is responsible to play role for reduction in fasting glucose. Adiponectin hormone reduces hepatic glucose output and lead towards increased peripheral insulin sensitivity through promotion of lipid oxidation process [43]. The reduction in blood glucose level might also be due to mechanisms based on substituting of fuel with increased glucose utilization and reduced fatty acid accessibility and enhancing the effect of insulin, the cycle involved with glucose-fatty acids could also be the reason [44].

The supplementation of Ω-3 PUFAs significantly decrease the body weight of PCOS rats fed SO, FO and F diets when compared it with rats fed PC diet. However, response of reduction in body weight was the same in rats fed different sources Ω-3 PUFAs. Vaughan et al. [45] had also found decrease in body weight of the PCOS subjects offering different sources of Ω-3 PUFAs in diets by improving metabolism and fat burning potential by the improvement of GLP-1 which is the ‘‘satiety’’ hormone, well recognized to do a major function in brain to comeback declining hunger and so energy intake [46, 47]. Ω-3 PUFAs fed by subjects also effect on leptin that reduce the appetite and effect on the production of the obese gene non-glycosylated protein, well known for its effect on dropping the body weight [48]. Bathena et al. [49] also proved that flaxseed had potential advantages of diminishing the fat preservation in the liver of hereditarily obese animals that ultimately might have effect on reducing body weight.

In this present study, Ω-3 PUFAs treatment advanced to disappearance of cysts. Higher concentrations of corpora lutea have been noticed which suggests that ovulation and estrous cyclicity was normal. Visible granulosa cell layer surrounded the oocytes which were developed within the ovary follicles at different development stages. Ovarian cortex appearance was also normal. Only a few corpus luteum and a large number of follicular cysts were seen in PCOS induced rats. The form of corpora lutea is considered as occurrence of ovulation. Earlier published studies have extensively described the numerous mechanisms for the impacts of Ω-3 PUFAs on ovaries and follicle structures. Ω-3 PUFAs have been observed as a stimulator of FSH which cause follicular growth, ability and development of follicular rupture and ovuation [50]. EPA and DHA are the two unique biological signaling factors that cause ovulation and enhance the flow of blood to the ovaries to promote follicles growth and increase ovarian weight [51]. The intake of Ω-3 PUFAs promotes the release of gonadotropin-releasing hormone (GnRH) primarily regulated by hypothalamic receptors of the reproductive system, which subsequently mediate the release of LH and FSH from pituitary glands [52]. A high intake of fish oil leads to a considerable rise in follicles development and ovulation and these actions depend on the hypothalamic-pituitary function [53]. Ω-3 PUFAs stimulated the follicogenesis process in ovaries, which may positively affect the efficiency of fertility [54]. The current study’s results were similar to the results of Manneras et al. [55] who exposed that the PCOS rat model induced by estradiol valerate had asymmetrical cycles. Their ovaries had huge, atretic antral follicles, follicular cysts with a thickened theca internal cell layer, and a small number of fresh corpora lutea, related to our current consequences. In this current study, the Ω-3 PUFAs supplementation significantly improved the corpora lutea concentration. Moreover, the corpora lutea level in relation to total follicle strength and antral follicle numbers was found higher which were noticed in line to Manni’s research [56]. The achievable clarification for the development of cyclicity is that reduced sympathetic activity which has a straight effect on the ovaries. This process also greatly affects the sex steroid synthesis pathways. It has been documented in research studies that physical exercise could lead towards reduced sympathetic nerve activity, regular menstrual frequency and lowers the sex steroid level in PCOS women [57]. Furthermore, the significant increase in granular layer thickness and corpora lutea area was seen in experimental groups treated with abundant Ω-3 PUFAs. The ratio of numbers of corpora lutea to total numbers of follicle was higher in treated groups. Lifestyle interference of human PCOS has been extensively accepted. Our experimental outcomes were also in agreement with the findings of Elaheh et al. [58] who observed that Ω-3 FAs could helpful to recover hyperemia and diminish hemorrhage in experimental PCO groups. Moreover, Ω-3 PUFAs play role to protect cells during injury and unfavorable conditions [59].

The digestibility of DM, CP, EE and CF was not change statistically in all rats fed diets containing Ω-3 PUFAs sources compared with NC and PC. No comprehensive work has been done regarding the direct effect of Ω-3 PUFAs on nutrient digestibility in PCOS rats. Digestibility results of the present study are in agreement with that of Smink et al. [60] who reported that omega fats have higher absorption efficiencies and consequently digestibility coefficients when compared with saturated fats. This is may be due to the fact that monounsaturated and polyunsaturated fats have the ability to form proper micelles which enhance the digestibility process, whereas saturated fats may have an irregular micelles formation because of their characteristic low polarity [61]. This characteristic of saturated fats would increase the viscosity of digesta in the gastrointestinal tract and subsequently decrease the digestion and absorption of saturated fats.

## Conclusions

This research work significantly contributed to the knowledge of Ω-3 PUFAs in PCOS by having positive impact in improving the productive and reproductive hormones, blood glucose, lipid profile, body weight, nutrient digestibility and ovarian morphology.

## Data Availability

The dataset supporting the conclusions of this article is included within the article.

## Abbreviations

ALA: α-Linolenic acid
CRD: Completely randomized design
DHA: Docosahexaenoic acid
EPA: Eicosapentaenois acid
FSH: Follicle stimulating hormone
GnRH: Gonadotropin-releasing hormone
HDL: High density lipoprotein
IM: Intramuscular
LDL: Low density lipoprotein
LH: Luteinizing hormone
Ω-3 PUFAs: Omega-3 polyunsaturated fatty acids
PCOS: Polycystic ovarian syndrome
